# Diagnosing serious infections in acutely ill children in ambulatory care (ERNIE 2 study protocol, part A): diagnostic accuracy of a clinical decision tree and added value of a point-of-care C-reactive protein test and oxygen saturation

**DOI:** 10.1186/1471-2431-14-207

**Published:** 2014-10-02

**Authors:** Jan Y Verbakel, Marieke B Lemiengre, Tine De Burghgraeve, An De Sutter, Dominique M A Bullens, Bert Aertgeerts, Frank Buntinx, on behalf of the ERNIE 2 collaboration

**Affiliations:** Department of General Practice, KU Leuven, Kapucijnenvoer 33, 3000 Leuven, Belgium; Department of Family Practice and Primary Health Care, Ghent University, De Pintelaan 185 6K3, 9000 Ghent, Belgium; Clinical Department of Paediatrics, University Hospitals Leuven, Leuven, Belgium; Paediatric Immunology, Department of Microbiology and Immunology, KU Leuven, Leuven, Belgium; Research Institute Caphri, Maastricht University, PB 313, Nl 6200 MD Maastricht, The Netherlands

**Keywords:** Child, Serious infections, Infant, Acute illness, C-reactive protein/analysis, Diagnostic accuracy, Safety netting, Point-of-care systems

## Abstract

**Background:**

Acute illness is the most common presentation of children to ambulatory care. In contrast, serious infections are rare and often present at an early stage. To avoid complications or death, early recognition and adequate referral are essential. In a recent large study children were included prospectively to construct a symptom-based decision tree with a sensitivity and negative predictive value of nearly 100%. To reduce the number of false positives, point-of-care tests might be useful, providing an immediate result at bedside. The most probable candidate is C-reactive protein, as well as a pulse oximetry.

**Methods:**

This is a diagnostic accuracy study of signs, symptoms and point-of-care tests for serious infections. Acutely ill children presenting to a family physician or paediatrician will be included consecutively in Flanders, Belgium. Children testing positive on the decision tree will get a point-of-care C-reactive protein test. Children testing negative will randomly either receive a point-of-care C-reactive protein test or usual care. The outcome of interest is hospital admission more than 24 hours with a serious infection within 10 days. Aiming to include over 6500 children, we will report the diagnostic accuracy of the decision tree (+/− the point-of-care C-reactive protein test or pulse oximetry) in sensitivity, specificity, positive and negative likelihood ratios, and positive and negative predictive values. New diagnostic algorithms will be constructed through classification and regression tree and multiple logistic regression analysis.

**Discussion:**

We aim to improve detection of serious infections, and present a practical tool for diagnostic triage of acutely ill children in primary care. We also aim to reduce the number of investigations and admissions in children with non-serious infections.

**Trial Registration:**

ClinicalTrials.gov Identifier: NCT02024282

## Background

Acute illness is the most common reason for encounter of children attending ambulatory care.

In a primary care setting, less than 1% of children assessed will have a serious illness [1]. The incidence of serious infections in children is assumed to be 5 to 10 times higher at the paediatric emergency department [2]. Febrile illness accounts for 20% of all paediatric ED visits [3].

Serious infections are rare in children in developed countries, but associated with considerable morbidity and mortality [4]. In Flanders, infectious diseases are responsible for 8.0% of all deaths in children under the age of one year, and for 13.6% of deaths in children aged 1 to 14 years [1, 5]. These numbers are comparable to death rates previously reported in the UK [6]. Serious infections in children are usually defined as sepsis, meningitis, pneumonia, pyelonephritis, gastroenteritis with dehydration, osteomyelitis, complicated abscess, viral respiratory infection with hypoxia, and cellulitis [7]. The mortality of meningococcal disease can be as high as 25% [8]. Besides this, approximately 7% of children who survive bacterial meningitis suffer from hearing loss [9].

Serious infections need to be distinguished from the vast majority of self-limiting viral conditions in children. Those few children with a serious infection can present at an early stage when the severity of the infection is not yet apparent [4]. At that point, their symptoms tend to mimic those of children with a non-serious illness. The rapid deterioration could cause a diagnosis to be missed at first contact, sometimes with severe consequences. Early recognition and adequate referral of serious infections are of vital importance to avoid complications. A faster and more accurate recognition of serious diseases could prevent unnecessary investigations, referrals, treatments and hospitalisations in children without serious illness, avoiding traumatic experiences for the child, parental concerns and health care expenditures.

### Assessment of serious infections

Clinicians use signs and symptoms to assess the probability of a serious infection and to decide on further management. To investigate the predictive value of these signs and symptoms, Van den Bruel et al. conducted a trial, which prospectively included almost 4000 children, resulting in a decision tree based on signs and symptoms [10]. This tree had a sensitivity and negative predictive value of nearly 100%. The risk of having a serious infection in children testing positive and thus indicating referral for further testing, however, was approximately 10%. If applied in clinical practice without caution, this decision tree could cause far too many children to be referred to hospital.

Vital signs are uncommonly measured in children in general practice [11]. If measurement of vital signs would become part of routine care, they could be useful in detecting serious infections in acutely ill children [12, 13]. A pulse oximetry, alongside other vital signs measurements has shown to differentiate children with serious infections from those with self-limiting infections in paediatric emergency care [13]. Validation of these results in low prevalence settings might aid clinicians to measure vital signs objectively.

A systematic review of the literature in all relevant databases identified the laboratory tests used to detect serious infections in febrile children in ambulatory settings [14, 15]. The most probable candidates for this purpose are CRP and procalcitonin (PCT). CRP can predict bacterial aetiology of community-acquired pneumonia [16]. PCT correlates with severity of urinary tract infections and sepsis and of community-acquired pneumonia in children [17, 18]. Despite these promising results, evidence is not yet conclusive and other tests may be valuable as well, urging for a large-scale trial.

POC tests are defined as laboratory and other services provided to patients at bedside. The physician has an immediate result and management can be adjusted accordingly. This makes them especially attractive in situations where a fast decision is warranted, such as urgent-access ambulatory care. They are minimally invasive, and thus relevant in paediatric care.

In diagnostic tests and clinical prediction rules a sensitivity of 100% is hard to achieve, because patients present at different stages in the evolution of their illness. At an early stage classic “red flag” features of serious illnesses tend to be absent. Safety netting is an integral part of the diagnostic process to deal with this situation. Neighbour and Almond et al. defined safety netting and sought clinical consensus about what safety netting should include: (I) the existence of uncertainty and how to communicate this to the patient or parent, (II) what exactly to look out for, (III) guidance on how exactly to seek further help and (IV) what to expect about time course [19–21]. Safety netting strategies and subsequent action should be evaluated on their effect on patient outcome, referral rate, antibiotic prescribing, and parental anxiety. In the ERNIE 2 trial, we will provide formal safety netting to a random sample of all children with a negative result on the decision tree, as described by Lemiengre et al. [22]. This includes a parent leaflet with instructions on how to detect and treat certain symptoms (e.g. fever), or a deterioration of the child’s condition, as well as when and how to re-consult the physician.

In this study, we aim to validate this decision tree in a new population and explore the added value of technological tests, such as Point-of-Care (POC) tests in diagnosing serious infection in acutely ill children in ambulatory care.

This trial is part of the ERNIE2-trial, which also comprehends a cluster randomised controlled trial to evaluate the effect of a POC CRP test and a brief intervention combined with a written safety net advice on additional testing, re-consultation and the antibiotic prescribing rate in acutely ill children not suspected of serious disease in primary care, as described by Lemiengre et al. [22].

## Methods

### Design

**Figure 1:**
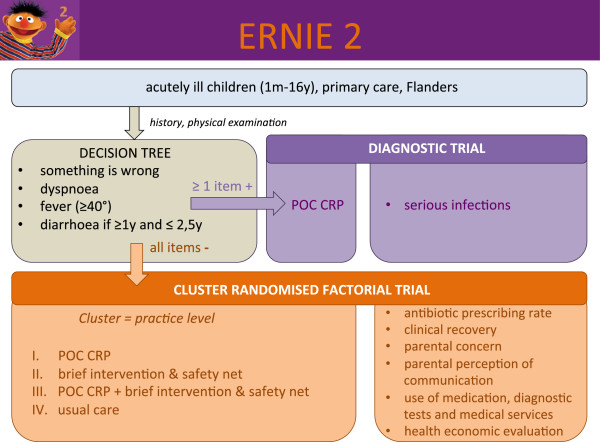
**Study design.** POC CRP, point-of-care C-reactive protein; m, month; y, year.

This is a prospective diagnostic accuracy study in ambulatory care (defined as general practice, paediatric outpatient clinics or emergency departments) identifying the diagnostic value of signs, symptoms and technological tests in diagnosing serious infections using hospital admission more than 24 hours for a serious infection as the main outcome measure (Figure 1).

### Participants

Children aged 1 month to 16 years, presenting to a family physician or paediatrician in Flanders, Belgium, with a new acute illness episode of maximum 5 days will be included consecutively. Children will be excluded if the acute illness is caused by merely traumatic or neurological conditions, intoxication, psychiatric or behavioural problem, or an exacerbation of a known chronic condition. If a physician includes the same child twice within 5 days, we will consider the second registration as a repeated measure and discard it subsequently from the analysis. Physicians will be instructed to recruit children consecutively during the inclusion period. If a physician includes less than five children over the study period, the assumption of consecutive inclusion is probably violated, and his or her results will subsequently be excluded from the analysis.

### Index tests

#### 5-stage decision tree

As part of a thorough history taking and physical examination, we will ask physicians to score the 5-stage decision tree, as developed by Van den Bruel et al. [10].

Children testing positive on this tree will always get a POC CRP test.

Children testing negative on this tree presenting to primary care will either get a POC CRP test, or usual care, depending on their per practice cluster randomisation, as described by Lemiengre et al. [22].

#### POC CRP test (finger stick)

We searched the literature in multiple databases and performed a survey of manufacturers, completed with personal contacts in order to identify tests that would be marketed shortly for acute infections in children in primary care. For use in children, important criteria of a feasible test are the sample volume (<5 μl) and the test duration (<5 minutes). Two point-of-care CRP tests (Afinion CRP test on the Afinion AS100 Analyzer, Alere, USA and CRP test on the LifeAssays™ Reader by LifeAssays, Sweden) met our criteria.

A pilot study was performed to determine the user-friendliness of these POC CRP devices. Only the Afinion CRP test on the Afinion AS100 Analyzer met our standards for user-friendliness. It is a user-friendly device, especially because a small drop of blood is enough to perform the test, what makes it perfect for use in children, and only two simple operations are needed to get the result (aspirating blood in the capillary, putting the cassette in the machine). In this pilot study, the analytical accuracy of the selected POC CRP device was also examined, demonstrating an agreement between the CRP test results on the Afinion AS100 Analyzer and the Cobas c702 (Roche Diagnostics, Switzerland) with a mean difference of 0.1% (95% CI: −17.6% to 17.4%) and perfect correlation (y = 1.01×-0.04) even at high CRP concentrations [23].

The Afinion™ CRP Test Cartridge consist of a 1.5 μL glass capillary to be filled with blood from a finger stick and a reagent container. First, the sample is automatically diluted with a liquid that also lyses the blood cells. Then, the sample mixture is aspirated through the membrane coated with anti-CRP antibodies, and all CRP in the sample is concentrated on this membrane. The conjugate solution containing anti-CRP antibodies labelled with ultra-small gold particles is then sucked through the membrane. The gold-antibody conjugate binds to the immobilized CRP on the membrane, which will turn red-brown. Excess gold-antibody conjugate is removed by a washing solution. The analyser measures the colour intensity of the membrane, which is proportional to the amount of CRP in the sample. The result is available within 4 minutes. The CRP measuring range is 5–150 mg/L.

We will instruct all physicians to properly perform the POC CRP test. For internal quality control, a low and a high control positive will be measured at regular intervals to confirm the efficacy and correct performance of the test according to the manufacturer’s instructions. The device distributor will provide technical assistance.

#### Pulse oximetry

All physicians will be asked to measure pulse oximetry by means of a paediatric finger pulse oximeter (CMS50QA, Contec™ Medical Systems, China), provided for this trial, measuring oxygen saturation and pulse rate. A small-scale pilot study was performed to determine the appropriate age requirements for the device (above 3 years of age) and agreement with a large-size pulse oximeter.

### Outcome measures

#### Primary outcome measure

1. Serious infections, defined as a hospital admission for more than 24 hours for any of these diagnoses:

- Sepsis (including bacteraemia) with a pathogenic bacteria isolated from a haemoculture as the reference standard
- Meningitis (viral or bacterial) with a positive lumbar puncture (pleocytosis in cerebrospinal fluid and identification of a bacteria or a virus) as the reference standard
- Abscess with a positive culture as the reference standard
- Pneumonia (viral or bacterial) with an infiltrate seen on chest x-ray as the reference standard
- Osteomyelitis (pathogens from bone aspirate as the reference standard, or if unavailable with a MRI or bone scan suggestive for osteomyelitis)
- Cellulitis (acute suppurative inflammation of the subcutaneous tissues)
- Gastro-enteritis with dehydration
- Complicated urinary tract infection (positive urine culture (>10^5^/ml pathogens of a single species) and systemic effects such as fever)
- Viral respiratory tract infections complicated by hypoxia (e.g. bronchiolitis)

If a serious infection occurs within 10 days after inclusion, it will be considered a consequence of the same illness episode. To ensure a firm definition of the outcome (a serious infection), the outcome will be measured through three strategies: (I) a thorough search of each child’s electronic medical record at the hospitals within the referral region of the participating FP or paediatrician, (II) the results from interviewing the physician who included the child in the study, and finally (III) the results from the diary filled out by parents after consulting the FP, mentioning the date the child is no longer ill.

#### Secondary outcome measure

1. Use of other diagnostic tests and medical services (including re-consultation).

### Sample size

Based on the binomial distribution, at an expected sensitivity of 97% with a minimal acceptable lower confidence limit of 85%, the minimal number of cases is 59, not taking into account the non-monotonic nature of power as a function of sample size due to discreteness of the binomial distribution [24]. The number of controls, derived through the formula N_controls_ = N_cases_ [(1-Prevalence)/Prevalence], [25] is 7316 at a prevalence of 0.8% and 6497 at a prevalence of 0.9%.

Considering the sample size calculation, we aimed to include 6500 children (in 88 general practices and 12 paediatric units), across Flanders, in urban and rural areas over a period of 12 months.

### Implementation

#### Recruitment of physicians

We will compose a letter with a short description of the research and the (dis)advantages of participating, with the option to request further information. This letter will be distributed by email to all FPs known to the research partners trough personal and professional contacts. Interested family physicians (FPs) will be contacted and visited by the researchers to inform them more extensively about the trial. Participating FPs will complete a short questionnaire about practice characteristics. For more details about this questionnaire, we refer to Lemiengre et al. [22]. After agreement to participate, all physicians will be visited and instructed on the inclusion process. At this point in time, we will inform the FPs about their allocation by the investigator. The step-by-step plan will be explained in detail. All FPs will get a demonstration to properly perform the POC CRP test.

We will ask the paediatricians involved in the project steering committee and their colleagues affiliated with a university hospital to participate in the study and include eligible children at the outpatient clinic, as well as the emergency department. We’ll ask them to contact other interested colleagues affiliated with hospitals across Flanders, with a workload of acutely ill children similar or higher compared to the university hospitals. After agreement to participate, a meeting with all participating paediatricians will be organised to explain the trial in detail. All paediatricians will get a demonstration to perform the POC CRP test.

Parents will be informed through posters in the waiting room, as well as flyers, with a short comprehensible description of the background, aims and requirements to participate. Physicians will inform every eligible child and their parent(s), delivering an information leaflet and asking formally to participate. Parents and children from the age of 12 will sign a written informed consent form, with a permission to access the hospital medical record in case of a possible admission. We will provide adjusted information leaflets and consent forms for minors below and above 12 years of age.

We will ask physicians to perform a thorough history taking and physical examination of every child, registering items based on experience from previous research and clinical consensus of an international team of clinicians and researchers, [26] such as measurement of the NICE traffic light system, the Yale Observation Scale, the 5-stage decision tree, and vital signs, such as a pulse oximetry [27–29].

The clinician will record the gathered data, preliminary diagnosis and planned actions (e.g. investigations, treatment or referral) on a case report form. Only parents of children attending a FP’s office will complete a booklet, containing 4 surveys and a follow up diary. For more details about this booklet and the related procedures, we refer to Lemiengre et al. [22].

We will ask parents to send a text message to the investigators on the day the child is no longer sick. These children will automatically enter a prize draw and prices will be awarded monthly amongst these children.

#### Follow-up

We intend to contact physicians at least on a monthly basis, via email, telephone or regular mail, as well as use occasional motivational gifts to remind them of this trial and encourage consecutive inclusions. A gift-savings system for physicians will be introduced with children toys for the doctor’s waiting room.

If a physician is clearly not including children or violating the in- or exclusion criteria of this study, the trial will be discontinued in this practice and replaced by a new motivated practice willing to participate, with similar practice type and region.

### Collaborating organizations

The research partners are:

- The Department of General Practice, Faculty of Medicine, KU Leuven, in collaboration with the Clinical Department of Paediatrics, UZ Leuven
- The Department of Family Practice and Primary Health Care, Faculty of Medicine and Health Sciences, Ghent University, in collaboration with the Department of Paediatrics, UZ Gent.

### Ethics

The protocol of this study was approved by the Ethical Review Board of the University Hospitals/KU Leuven, under reference ML8601. All children’s parents are requested to provide written informed consent. As soon as all hospitals within the referral region of all participating FPs are known, these centres will be submitted for formal approval by the coordinating and the local ethical review boards.

### Statistical analysis

The data will be stored and analysed at two locations, KU Leuven and Ghent University, using Excel (Microsoft Corporation, Redmond, USA), Stata software (version 11.2; Stata Corp., College Station, TX, USA), SPSS (version 20; SPPS Inc., Chicago, Illinois, USA) and QSR NVivo version 10 (QSR International Pty Ltd, Melbourne, Australia).

The diagnostic accuracy of the 5-stage decision tree will be tested and reported in sensitivity, specificity, positive and negative likelihood ratios, and positive and negative predictive values with their 95% confidence intervals (CI). Whenever possible, Receiver Operating Characteristic (ROC) curves will be plotted in order to identify the optimal cut-off value, as well as forest and dumbbell plots of positive and negative predictive value for presentational purposes [4]. The value of the POC CRP test and the pulse oximetry will each be added to the 5-stage decision tree and compared to the results of the 5-stage decision tree alone, to determine the added value of the selected technology. New diagnostic algorithms will be constructed through Classification and Regression Tree and multiple logistic regression analysis, while accounting for all clinically plausible interactions, resulting in a new multivariable model. Goodness-of-fit and discrimination testing will be performed, as well as regression diagnostics to look for individuals with excessive influence on the model.

We will compare the use of additional diagnostic tests, medical services and re-consultation between children who always get a POC CRP test, and children who only get a POC CRP test after a positive result on the decision tree. We will balance the costs and benefits of this intervention.

## Discussion

Feverish illness is the most common reason for encounter of children attending ambulatory care. In contrast, serious infections are rare in children in developed countries, but associated with considerable morbidity and mortality.

Those few children with a SI can present at an early stage when the severity of the infection is not yet apparent. A faster and more accurate recognition of serious diseases could prevent unnecessary investigations, referrals, treatments and hospitalisations in children without serious illness. On the other hand, children with apparent signs of a life-threatening serious infection should immediately be referred to or seen at the ED or an urgent-access paediatric clinic. Unnecessary delay of adequate treatment and management decisions should be avoided in these children.

Signs and symptoms are the first information to support clinical decision making in primary care [30]. Parental concern is an important predictor of SI, as well as the clinician’s feeling that “something is wrong” (gut feeling) [10]. Other red flags, such as cyanosis, rapid breathing, poor peripheral circulation, meningeal irritation and petechial rash have been shown to increase the likelihood of a serious infection in ill children [4].

Blood tests are only rarely performed in acutely ill children in primary care, due to the need to make management decisions prior to the availability of test results. Very little research has been performed in ambulatory care and none of it in primary care specifically. However, a role has been put out for CRP and procalcitonin to rule out serious infections [15].

POC tests enable physicians to adjust their management according to the immediate test results. They are minimally invasive, and thus relevant in paediatric care.

Research is needed to further inform clinicians and parents. Triage, face-to-face assessment in primary care, as well as evaluating clinical features, laboratory tests and safety netting, needs to be further examined [30].

To our knowledge, this is the first large-scale trial, investigating the (added) value of POC CRP in addition to clinical features in identifying serious infections in acutely ill children in ambulatory care, including general practice, paediatric outpatient clinics and hospital emergency departments.

Currently, no reliable cut-offs for CRP are known to differentiate between viral and bacterial causes of acute infection in children, nor for referral or discriminating between serious infections and self-limiting disorders. Therefore, physicians did not receive any guidance on the interpretation of the CRP results. Furthermore, we did not impose any restriction on the physicians’ care concerning treatment, additional testing, referral or hospital admission.

The main challenges of this project will be recruitment of study participants, avoidance of non-consecutive inclusions, and verification of the outcome measures. To ensure sufficient recruitment, several reminders through various ways and small presents for all participating physicians will be provided, endorsed by a personal approach. We also made sure to use very small finger-stick devices which cause only very limited pain or discomfort to the children, which were subsequently rewarded with a small present (finger-puppet). If a physician includes less than five children over the study period, the assumption of consecutive inclusion is probably violated, and his or her results will be excluded from the analysis.

To ensure a firm definition of the serious infections, the outcome was measured through three different strategies, which is the clinically sensible thing to do.

In diagnostic accuracy studies with low prevalence of the target condition, it is often difficult to calculate the required sample size with sufficient power. A recent developed nomogram to calculate sample size in diagnostic studies was not useful, because prevalences below 1% were not computed in the nomogram [31].

We aim to improve detection of SI, and present a practical tool for diagnostic triage of these children in primary care. We also aim to reduce the number of investigations and admissions in children with non-serious infections.

## Abbreviations

SI: Serious infections
POC: Point-of-care
CRP: C-reactive protein
PCT: Procalcitonin
FP: Family physician
ED: Emergency department
ROC: Receiver operating characteristic.
